# Design and Fabrication of a Miniaturized GMI Magnetic Sensor Based on Amorphous Wire by MEMS Technology

**DOI:** 10.3390/s18030732

**Published:** 2018-03-01

**Authors:** Jiawen Chen, Jianhua Li, Yiyuan Li, Yulong Chen, Lixin Xu

**Affiliations:** School of Mechatronical Engineering, Beijing Institute of Technology, Beijing 100081, China; 2120160268@bit.edu.cn (J.C.); lyy510@bit.edu.cn (Y.L.); 2220160063@bit.edu.cn (Y.C.); lxxu@bit.edu.cn (L.X.)

**Keywords:** amorphous wire, magnetic sensor, micro coils, MEMS, miniaturization

## Abstract

A miniaturized Co-based amorphous wire GMI (Giant magneto-impedance) magnetic sensor was designed and fabricated in this paper. The Co-based amorphous wire was used as the sense element due to its high sensitivity to the magnetic field. A three-dimensional micro coil surrounding the Co-based amorphous wire was fabricated by MEMS (Micro-Electro-Mechanical System) technology, which was used to extract the electrical signal. The three-dimensional micro pick-up coil was designed and simulated with HFSS (High Frequency Structure Simulator) software to determine the key parameters. Surface micro machining MEMS (Micro-Electro-Mechanical System) technology was employed to fabricate the three-dimensional coil. The size of the developed amorphous wire magnetic sensor is 5.6 × 1.5 × 1.1 mm^3^. Helmholtz coil was used to characterize the performance of the device. The test results of the sensor sample show that the voltage change is 130 mV/Oe and the linearity error is 4.83% in the range of 0~45,000 nT. The results indicate that the developed miniaturized magnetic sensor has high sensitivity. By testing the electrical resistance of the samples, the results also showed high uniformity of each device.

## 1. Introduction

With the development of high precision magnetic detecting technology, there is an increasing interest in magnetic sensors with ultra-high sensitivity. Compared with conventional magnetic sensors such as Hell sensors, fluxgate sensors, AMR (Anisotropic Magnetoresistance) sensors or GMR (Giant Magnetoresistance) sensors, the GMI (Giant magneto-impedance) magnetic field sensor has the advantages of high sensitivity, fast response and low power consumption [1,2,3,4]. It has been widely used in the field of weak magnetic measurement and other detection fields.

Co-based amorphous wire has a higher impedance change rate than amorphous ribbon and amorphous film, so it is the most suitable sensitive material for GMI (Giant magneto-impedance) magnetic sensor [5,6,7]. The excellent GMI (Giant magneto-impedance) effect of Co-based amorphous wire may be related to the high transverse permeability caused by the transversely oriented domain configuration [8,9]. Wei et al. reported a GMI (Giant magneto-impedance) magnetic sensor made up of amorphous wire, insulation layer connector, conducting layer, outer insulation layer, checking coil and terminal block [10]. The conducting layer is copper or aluminum made by electroplating and the pick-up coil was wounded 30 circles with wires of diameter less than 200 µm. The amorphous wire and conducting layer are connected by the connector and made on the same axis. Nie et al. developed a differential-type integrating GMI (Giant magneto-impedance) magnetic sensor [11]. The offset coil of sensor is made up of a 0.20-mm-diameter enameled wire wrapping for about 200 turns around the amorphous wire. In order to reduce the small stress effect to the sensor, the amorphous wires are fixed on the PCB by elastic silicon rubber. The GMI (Giant magneto-impedance) magnetic sensor exhibits a linearity error about 0.92% FS in the measuring range of ±2.0 Oe, and the sensitivity can achieve about 748 mV/Oe. Gudoshnikov et al. designed a magnetometer based on the off-diagonal GMI (Giant magneto-impedance) effect in Co-rich glass-coated microwire [12]. The sensing element of the magnetometer is a 10 mm long piece of CoFeNiBSiMo micro wire with a small pick-up coil of 85 turns wound around the micro wire. The magnetometer is capable of measuring a narrow range of magnetic fields ±3.5 µT, while the range up to ±250 µT when using feedback circuit. The sensitivity of magnetometer is 30 V/T at *I* = 0.22 mA. However, the above amorphous wire sensors are still large and not easy to produce with batch fabrication. 

Magnetic sensors fabricated by MEMS (Micro-Electro-Mechanical System) technology have also been widely studied to reduce the sensor size. Ye et al. fabricated a Co-based amorphous ribbon with a tortuous structure by MEMS (Micro-Electro-Mechanical System) technology, which can be considered as a good candidate to fabricate the GMI (Giant magneto-impedance) based sensor with excellent performance [13]. The maximum longitudinal GMI (Giant magneto-impedance) ratio of the tortuous shape ribbon is 193.7%, the magnetic field sensitivity is 17.4%/Oe, and the dimension of the ribbon is 4400 μm (width) × 10,000 μm (length), respectively. Elsayed et al. presented a combined magnetometer/accelerometer sharing a single surface micromachined structure [14]. The device was fabricated using a low-temperature surface micromachining technology, which was adapted for above-IC integration on standard CMOS substrates. The magnetic field and acceleration sensitivities of the device were measured to be 1.57 pF/T and 1.02 fF/g. Guo et al. reported a novel micro-fluxgate sensor based on a double-layer magnetic core of a FeCoB-based amorphous ribbon [15]. The micro-fluxgate sensor was fabricated via MEMS (Micro-Electro-Mechanical System) technology combined with chemical wet etching. The resulting sensor exhibited a sensitivity of 1985 V/T, and a wide linearity range of ±1.05 mT. Long et al. presented a novel MEMS (Micro-Electro-Mechanical System) magnetic sensor based on fiber-optic detection [16]. The magnetic field was detected by measuring the tilting motion of a mechanical suspension with a permanent magnet attached. The magnetic sensor was fabricated by using a bulk micromachining process and assembled with the permanent magnet manually. This magnetic sensor realizes a sensitivity of 2.86 mV/µT for the in-plane magnetic field and 6.57 mV/µT for the out-of-plane. The above amorphous wire magnetic sensors and MEMS (Micro-Electro-Mechanical System) magnetic sensors are summarized in Table 1.

Though many investigations on magnetic sensors have been performed, few studies have been carried out on the miniaturization of the GMI (Giant magneto-impedance) sensor based on amorphous wire. In particular, the pick-up coil was wound by automatic winding machine or artificial winding, which would make the amorphous wire bend and induce stress [17,18,19,20]. Besides, the amorphous wire was generally electrically connected by welding technology such as soldering, resistance welding or ultrasonic welding. Because the amorphous wire is difficult to weld, the electrical resistance shows poor uniformity and consistency. These two problems result in poor performance and complicate the batch fabrication of the sensors.

In this paper, a miniaturized amorphous wire based GMI (Giant magneto-impedance) sensor was proposed. The structure and parameters of micro pick-up coil were first determined by modeling and simulating. Then the amorphous wire integrated into the micro three-dimensional coils by means of MEMS (Micro-Electro-Mechanical System) technology. Finally, the characteristics of the fabricated GMI (Giant magneto-impedance) sensor were measured by magnetic field calibration system.

## 2. Device Design

### 2.1. Sensor Miniaturization Design

**Figure 1:**
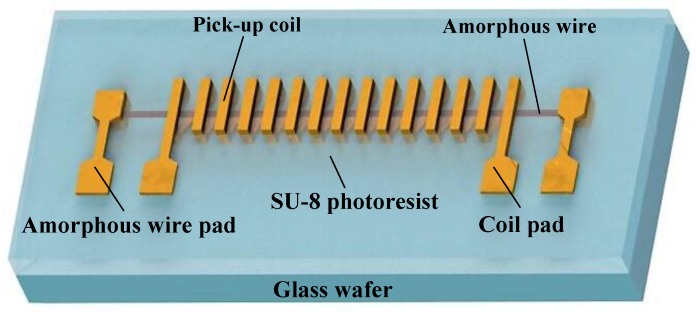
The three-dimensional model of sensor sensitive probe.

The overall design of a miniaturized GMI (Giant magneto-impedance) sensor is shown in Figure 1. The Co-based amorphous wire and micro signal pick-up coil are integrated on the glass wafer. Glass is used as the device-supporting layer and micro amorphous wire is the sense element. A micro pick-up coil is designed to wrap the micro amorphous wire for signal extraction. The entire device is coated with a layer of SU-8 negative photoresist, which is used as the supporting layer of the micro-coil upper layer. Electrical connection pads are on the surface of the SU-8 layer.

### 2.2. Micro Pick-up Coil Design

**Figure 2:**
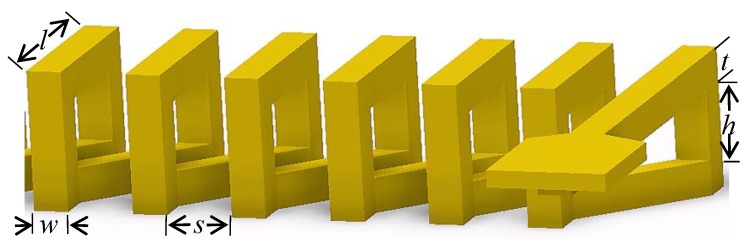
The geometric structure parameters diagram of the micro-inductor coil.

The signal pick-up coil consists of four parts: the bottom coil, the pillar, the dielectric layer and the top coil. According to the sensor working principle, the pick-up coil can be equivalent to an inductor. The change of the external magnetic field causes the change of the amorphous wire impedance, which creates the electromotive force (EMF) of the coil. The amplitude of the induced EMF across the coil can be expressed as:

(1) $$E_{c}=NL_{c}\frac{dI_{c}}{dt}$$

where *N* is the turns of the pick-up coil, *L_c_* is the coil inductance, *I_c_* is the induced current across the coil. The inductance of the coil is defined as Equation (2):

(2) $$L_{c}=\frac{d\phi }{dI_{c}}$$

where *ϕ* is the magnetic flux through a single turn coil. Equation (1) shows that *E_c_* can be improved by increasing the coil inductance. The performance of the micro-inductor at high frequency is not only related to the substrate and the coil material, but also to the geometric parameters of the micro-inductor coil. Figure 2 is the geometric structure parameters diagram of the micro-inductor coil. The length of the top (bottom) coil wire is *l*, the width of the coil wire is *w*, the thickness of the coil wire is *t*, the horizontal space between adjacent metal wires is *s*, the width of the pillar is equal to the coil wire width *w* and the height of the pillar is *h*.

**Figure 3:**
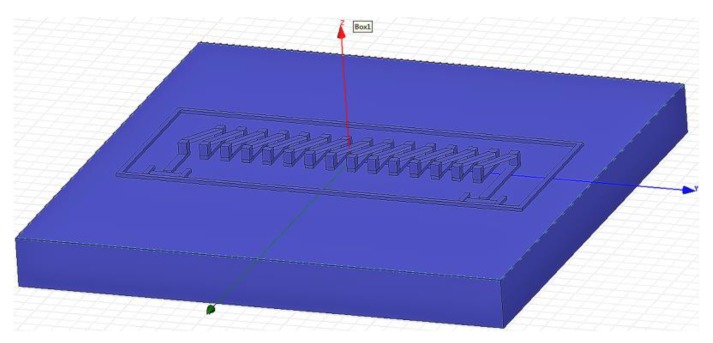
Coil structure model in HFSS (High Frequency Structure Simulator). The coil parameters in this model can be manually adjusted within a certain range.

The structure model of the coil is established in HFSS (High Frequency Structure Simulator) software, as shown in Figure 3. By changing the wire length, wire width, wire space and the pillar height of the pick-up coil, respectively, the optimum parameters are achieved to get the maximum inductance value.

**Figure 4:**
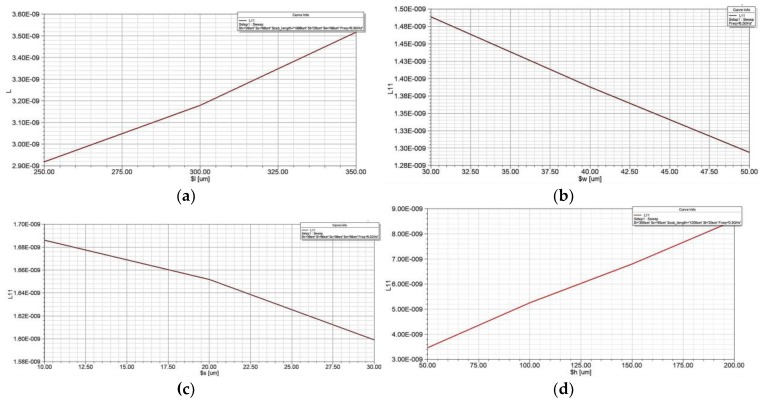
The effect of coil parameters on the inductance-factor. (**a**) The effect of wire length on the inductance-factor; (**b**) The effect of wire width on the inductance-factor; (**c**) The effect of wire space on the inductance-factor; (**d**) The effect of pillar height on the inductance-factor.

Figure 4 shows the simulation results of the effect of the different design parameters on the coil inductance. It can be concluded from Figure 4 that the inductance of the coil is positively correlated with the wire length and the pillar height, and it is contrary to the trend of wire width and wire space. Therefore, the inductance of the pick-up coil can be improved by increasing the wire length and the pillar height. The wire width and the wire space should not be too large, otherwise they will reduce the performance of micro-coil and increase the chip area occupied by the coil. Based on the above discussion and the requirements of miniaturization, the parameters of the three-dimensional micro pick-up coil are set to the values shown in Table 2.

## 3. Device Fabrication

In this paper, positive photoresist is employed as sacrificial material to fabricate the bottom line of the micro pick-up coil and the pillars. SU-8 is used as the supporting layer of the upper layer of the micro pick-up coil. The detailed process flow is described as follows: the bottom line and the pillar of the three-dimensional micro structural coil are first processed. Then the amorphous wire is placed into the micro structural coil on the chip. Finally, the thick SU-8 negative photoresist is spin coated to fabricate the following upper layer of the micro pick-up coil and electrical connections of the pads to the amorphous wire.

**Figure 5:**
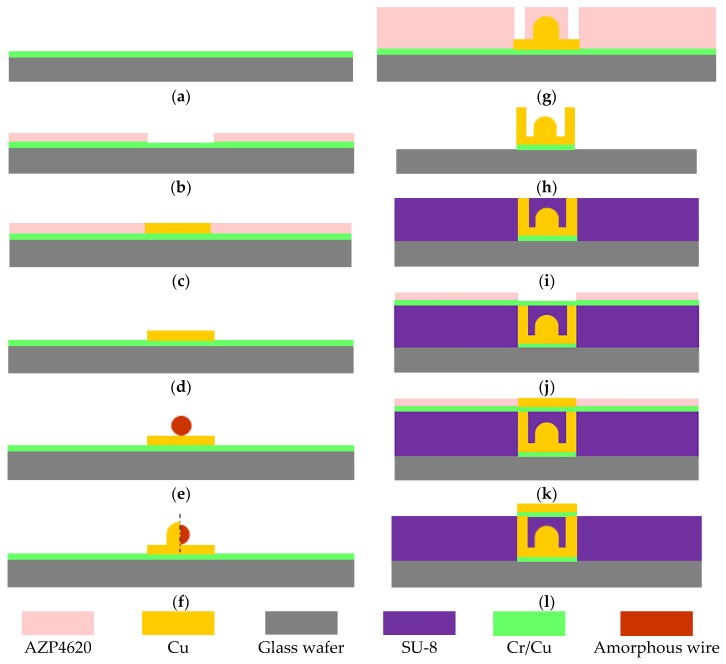
Fabrication process flow of amorphous wire GMI (Giant magneto-impedance) magnetic sensor. (**a**) Sputtering a Cr/Cu seed layer on the front side of the glass wafer. (**b**) Spin coating AZP4620 photoresist, then exposing to form the pattern of the bottom lines of the coil. (**c**) Electroplating copper to form the bottom lines of the coil. (**d**) Removing the photoresist. (**e**) Fixing the amorphous wire above the bottom lines of the coil. (**f**) Electroplating copper at both ends of the amorphous wire. (**g**) Spin coating AZP4620 photoresist, drying, exposing and developing to form the pattern of the pillars of the coil. (**h**) Electroplating copper to form the pillars of the coil. (**i**) Spin coating SU-8 negative photoresist, grinding and polishing to expose the micro Cu pillars. (**j**) Spin coating AZP4620 photoresist to form the pattern of the upper layer of the coil. (**k**) Electroplating copper in the pattern to form the upper layer of the coil. (**l**) Removing the photoresist, etching the seed layer.

The detailed fabrication process flow is shown in Figure 5: (a) Sputtering a Cr (600 Å)/Cu (2000 Å) seed layer by magnetron sputtering on the front side of the glass wafer, sputtering conditions: background vacuum is 1.00 × 10^−6^ torr, sputtering power of Cr is 1000 W and pressure of Ar is 5 mtorr, sputtering power of Cu is 2000 W and pressure of Ar is 8 mtorr. (b) Spin coating 5 µm AZP4620 photoresist (temperature of baking photoresist for 98 °C, time is 3 min), then exposing (using the alignment mark on the back of the substrate for UV lithography, hard contact, the light intensity is 7.7 MW/cm^2^, the time is 170 s and developing for 3 min to form the pattern of the bottom lines of micro pick-up coil. (c) Electroplating copper to form the bottom lines of the micro pick-up coil. (d) Removing the photoresist with acetone to expose the seed layer. (e) Fixing the amorphous wire above the bottom lines of the micro pick-up coil by adhesive. This operation is performed manually under microscope. (f) Electroplating copper at both ends of the amorphous wire for the electrical connections. (g) Spin coating AZP4620 photoresist, then drying, exposing and developing to form the pattern of the pillars of the micro pick-up coil. (h) Electroplating copper to form the pillars of the micro pick-up coil, then removing the photoresist and the seed layer. (i) Spin coating SU-8 negative photoresist, then grinding and polishing to expose the micro Cu pillars. (j) Spin coating AZP4620 photoresist, exposing and developing to form the pattern of the upper layer of the micro pick-up coil. (k) Electroplating copper in the pattern to form the upper layer of the micro pick-up coil. (l) Removing the photoresist with acetone, etching the seed layer. Finally, the miniaturized amorphous wire GMI (Giant magneto-impedance) magnetic sensor is obtained.

**Figure 6:**
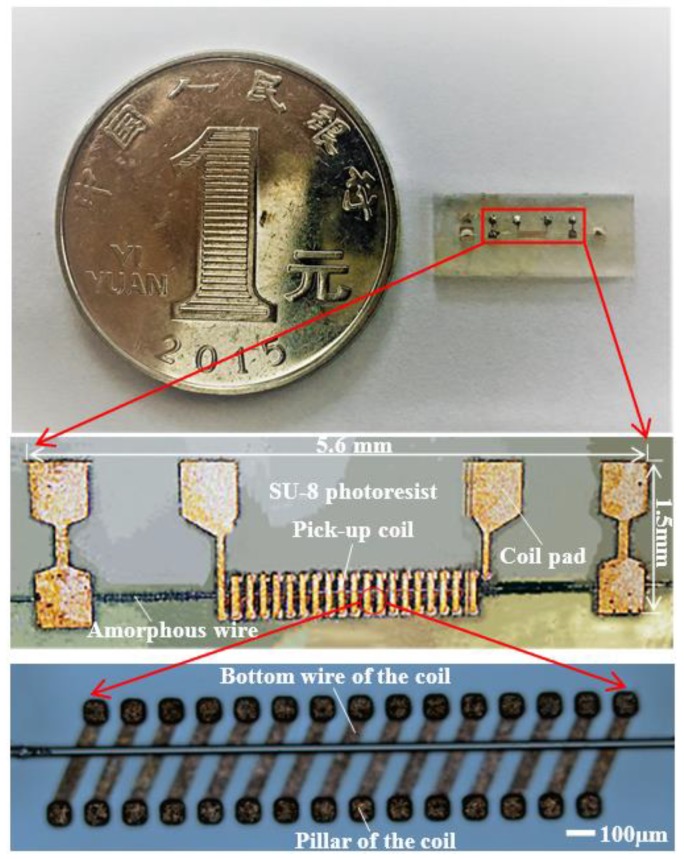
Fabricated amorphous wire GMI (Giant magneto-impedance) magnetic sensor probe. The bottom wire of coil is placed at an angle relative to the amorphous wire.

The size of the fabricated amorphous wire magnetic sensor is about 5.6 × 1.5 × 1.1 mm^3^, as shown in Figure 6. According to Table 1, the length of traditional amorphous wire magnetic sensor is 10 mm and the diameter of the pick-up coil is 50 mm. Therefore, the process has significantly reduced the size of the amorphous wire magnetic sensor.

## 4. Device Testing and Results 

**Figure 7:**
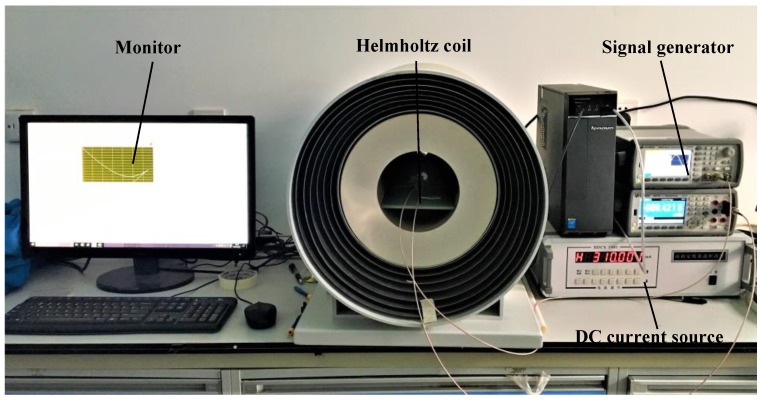
Experimental set up to measure the output of the magnetic field sensor. The outside of the Helmholtz coil is an 8-layer metal shield that allows the sensor to be undisturbed by the external magnetic field.

**Figure 8:**
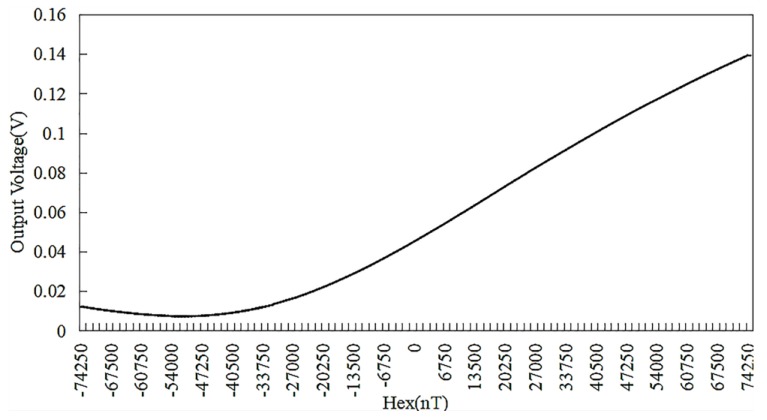
Output characteristic curve of the amorphous wire magnetic sensor sample in the range of the −74,250 nT~74,250 nT.

A miniaturized amorphous wire GMI (Giant magneto-impedance) sensor sample, the pick-up coil of which has 30 turns, was measured by magnetic field calibration system, as shown in Figure 7. The experimental setup mainly includes: electromagnetic shielding tube, Helmholtz coil, DC current source, signal generator. When testing the sample, it is placed in the middle of the electromagnetic shielding tube. The current of the DC current source can be varied between −1 and 1 A with 0.01 increment to produce a calibrated uniform magnetic field. The frequency of signal generator is set to 8 MHz, the amplitude is 5 V and the offset is 2.5 V. The output of the amorphous wire GMI (Giant magneto-impedance) sensor is displayed on the computer connected to the shielding tube. According to three measurements of the sensor output, the output characteristic curve is drawn, as shown in Figure 8. The curve reveals the relationship between the output voltage of the amorphous wire GMI (Giant magneto-impedance) sensor and the external magnetic field: the voltage varies linearly with the magnetic field in the range of 0~45,000 nT. The characteristics of the GMI (Giant magneto-impedance) sensor are also obtained: the voltage change rate is 130 mV/Oe, the linearity error is 4.83%.

**Figure 9:**
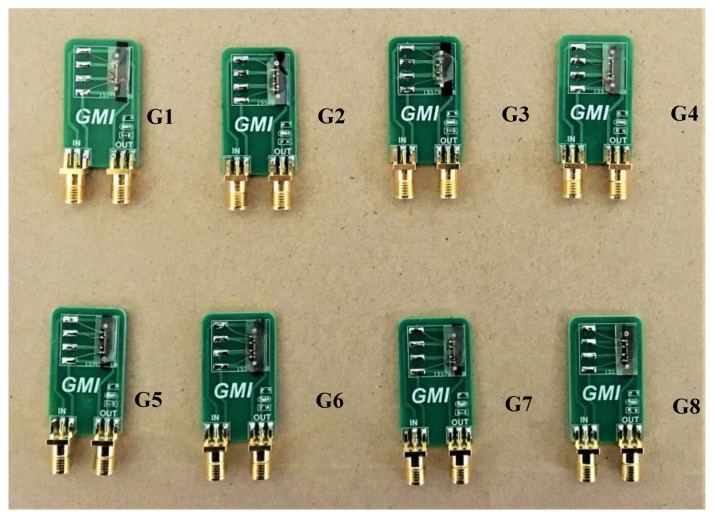
Eight sensors in the same batch of products were selected and numbered G1–G8 to test the consistency of MEMS (Micro-Electro-Mechanical System) technology.

**Figure 10:**
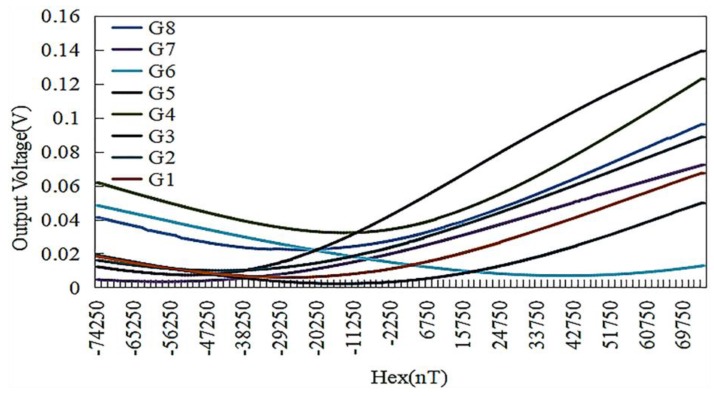
The consistency curve of the sensor output.

In order to verify the feasibility of the process and product uniformity, we further selected a group of eight magnetic sensors to test their impedance and output characteristics, which were labeled as G1–G8 (Figure 9). Table 3 shows the impedance values of the sensor coils and amorphous wires and the voltage values in the geomagnetic field, respectively. The consistency curve of the sensor output is plotted, as shown in Figure 10. It can be seen from Table 3 and Figure 10 that the maximum impedance difference of amorphous wire is 0.45 Ω. The output curves also show that the sensor performances except G6 are roughly the same, indicating the good uniformity and consistency of the fabricated amorphous wire sensors. In particular, G6 does not show good characteristics accordance, which indicates that further studies need to be carried out.

## 5. Conclusions

A Co-based amorphous wire miniaturized magnetic sensor and its fabrication process are presented in this paper. The size of the processed magnetic sensor is 5.6 × 1.5 × 1.1 mm^3^. A voltage change rate of 130 mV/Oe and a linearity error of 4.83% was achieved in the range of 0~45,000 nT. This amorphous wire GMI (Giant magneto-impedance) magnetic sensor meets the design requirements of miniaturization and high sensitivity and improves the processing uniformity and consistency of the device. Furthermore, the technique of SU-8 negative photoresist as a device support layer can also be used to prepare other three-dimensional suspended microstructures. For its good performance, the amorphous wire GMI (Giant magneto-impedance) magnetic sensor could have potential applications in week magnetic fields such as spacecraft navigation, intelligent traffic control and medical laboratory.

## Figures and Tables

**Table 1:** Comparison of various magnetic sensors.

Type	Size	Sensitivity	Fabrication Method
Magnetic detector with conducting layer	amorphous wire diameter: 30 µm, length: 3 mm; pick-up coil diameter: 200 µm, turns: 30.	65 mV/Oe in the range of −3 Oe~+3 Oe	welding, artificial winding
Differential-type integrating GMI (Giant magneto-impedance) magnetic sensor	amorphous wire diameter: 125 µm, length: 20 mm; pick-up coil diameter: 0.2 mm, turns: 200.	748 mV/Oe in the range of −2 Oe~+2 Oe	welding, artificial winding
Magnetometer based on the off-diagonal GMI (Giant magneto-impedance) effect	amorphous wire diameter: 10.7 µm, length: 10 mm; pick-up coil diameter: 50 mm, turns: 85.	Measuring range: ±250 µT	welding, artificial winding
Magnetometer/Accelerometer	1 × 1 mm^2^	magnetic field sensitivities: 1.57 pF/T Acceleration sensitivities: 1.02 fF/g	MEMS (Micro-Electro-Mechanical System)
Micro-fluxgate sensor with double-layer magnetic core	7.3 × 2.7 mm^2^	1985V/T in the range of −1.05 mT~+1.05 mT	MEMS (Micro-Electro-Mechanical System)
Torsion MEMS (Micro-Electro-Mechanical System) magnetic sensor with permanent magnet	3.09 × 3.09 mm^2^	in-plane magnetic field: 2.86 mV/µT out-of-plane magnetic field: 6.57 mV/µT	MEMS (Micro-Electro-Mechanical System)

**Table 2:** The parameters values of the pick-up coil.

Parameter of the Pick-up Coil	Value (µm)
wire length, *l*	350
wire width, *w*	30
wire space, *s*	20
pillar height, *h*	100

**Table 3:** The consistence of impedance and voltage output of sensors.

Number	Amorphous Wire Resistance (Ω)	Coil Resistance (Ω)	The Maximum Voltage (V)	The Minimum Voltage (V)
G1	12.386	0.771	210	23
G2	12.40	1.10	172	20
G3	12.280	1.31	182	32
G4	12.574	0.782	230	26
G5	12.425	0.850	144	16
G6	12.256	1.030	240	28
G7	12.113	1.236	224	30
G8	12.706	0.676	122	18
